# 3-Meth­oxy­carbonyl-1-methyl­pyrazinium tetra­chlorido(pyrazine-2-carboxyl­ato-κ^2^ *N* ^1^,*O*)stannate(IV)

**DOI:** 10.1107/S1600536811001929

**Published:** 2011-01-22

**Authors:** Ezzatollah Najafi, Mostafa M. Amini, Seik Weng Ng

**Affiliations:** a Department of Chemistry, General Campus, Shahid Beheshti University, Tehran 1983963113, Iran; b Department of Chemistry, University of Malaya, 50603 Kuala Lumpur, Malaysia

## Abstract

In the reaction of pyrazine-2-carb­oxy­lic acid and stannic chloride in methanol, one equivalent of the carb­oxy­lic acid is methyl­ated at the 4-amino site and is also esterified, yielding the title salt, (C_7_H_9_N_2_O_2_)[SnCl_4_(C_5_H_3_N_2_O_2_)]. The Sn^IV^ atom in the anion is *N*,*O*-chelated by a pyrazine-2-carboxyl­ate in a *cis*-SnNOCl_4_ octa­hedral geometry.

## Related literature

For related organotin structures, see: Ma *et al.* (2004 ▶).
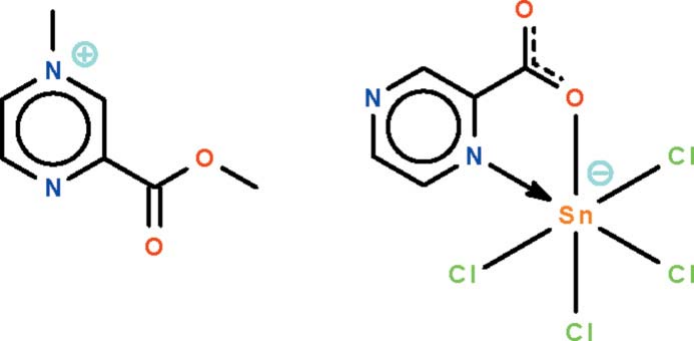


## Experimental

### 

#### Crystal data

(C_7_H_9_N_2_O_2_)[SnCl_4_(C_5_H_3_N_2_O_2_)]
*M* *_r_* = 536.75
Monoclinic, 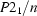

*a* = 7.0655 (2) Å
*b* = 26.7603 (7) Å
*c* = 9.5220 (2) Å
β = 94.554 (2)°
*V* = 1794.69 (8) Å^3^
*Z* = 4
Mo *K*α radiation
μ = 2.05 mm^−1^
*T* = 100 K
0.30 × 0.25 × 0.20 mm

#### Data collection

Agilent SuperNova Dual diffractometer with an Atlas detector
Absorption correction: multi-scan (*CrysAlis PRO*; Agilent Technologies, 2010 ▶) *T*_min_ = 0.579, *T*_max_ = 0.685
8726 measured reflections
3964 independent reflections
3582 reflections with *I* > 2σ(*I*)
*R*_int_ = 0.024

#### Refinement

*R*[*F* ^2^ > 2σ(*F* ^2^)] = 0.025
*wR*(*F* ^2^) = 0.059
*S* = 1.02
3964 reflections
228 parameters
H-atom parameters constrained
Δρ_max_ = 0.58 e Å^−3^
Δρ_min_ = −0.71 e Å^−3^

### 

Data collection: *CrysAlis PRO* (Agilent Technologies, 2010 ▶); cell refinement: *CrysAlis PRO*; data reduction: *CrysAlis PRO*; program(s) used to solve structure: *SHELXS97* (Sheldrick, 2008 ▶); program(s) used to refine structure: *SHELXL97* (Sheldrick, 2008 ▶); molecular graphics: *X-SEED* (Barbour, 2001 ▶); software used to prepare material for publication: *publCIF* (Westrip, 2010 ▶).

## Supplementary Material

Crystal structure: contains datablocks global, I. DOI: 10.1107/S1600536811001929/si2325sup1.cif

Structure factors: contains datablocks I. DOI: 10.1107/S1600536811001929/si2325Isup2.hkl

Additional supplementary materials:  crystallographic information; 3D view; checkCIF report

## Figures and Tables

**Fig. 1.**
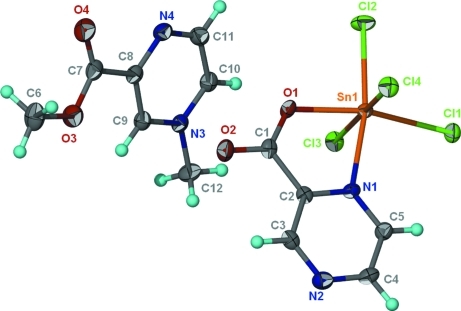
Thermal ellipsoid plot (Barbour, 2001) of [C7H9N2O2]+ [SnCl4(C5H3N2O2)]- at the 70% probability level; hydrogen atoms are drawn as spheres of arbitrary radius.

## supplementary crystallographic information

### Comment

The direct synthesis of a potentially chelating amino-carboxylic acid with stannic tetrachloride has not been reported. Pyrazine-2-carboxylic acid yields a number of derivatives with organotin compounds; these are either synthesized by condensing the amino-carboxylic acids with an organotin oxide/hydroxide or by reacting the amino-carboxylic acids with an organotin chloride in the presence of a proton abstractor. With the latter route, the product may be an organostannate in which the pyridine-2-carboxylate chelates to the chlorine-bonded tin atom (Ma *et al.*, 2004). In the reaction of pyrazine-2-carboxylic acid and stannic chloride in methanol, one equivalent of the carboxylic acid is methylated at the 4-amino site and is also esterified to yield the salt, [C_7_H_9_N_2_O_2_]^+^ [SnCl_4_(C_5_H_3_N_2_O_2_)]^-^ (Scheme I, Fig. 1). The tin atom in the anion is *N*,*O*-chelated by a pyrazine-2-carboxylate in a *cis*-SnNOCl_4_ octahedral geometry.

### Experimental

Stannic chloride pentahydrate 0.35 g, 1 mmol) and pyrazine-2-carboxylic acid (0.13 g, 1 mmol) were loaded into a convection tube; the tube was filled with dry methanol and kept at 333 K. Colorless crystals were collected from the side arm after several days.

### Refinement

Carbon-bound H-atoms were placed in calculated positions [C—H 0.95 to 0.98 Å, *U*_iso_(H) 1.2 to 1.5*U*_eq_(C)] and were included in the refinement in the riding model approximation.

### Crystal data

**Table:**

(C_7_H_9_N_2_O_2_)[SnCl_4_(C_5_H_3_N_2_O_2_)]	*F*(000) = 1048
*M**_r_* = 536.75	*D*_x_ = 1.987 Mg m^−^^3^
Monoclinic, *P*2_1_/*n*	Mo *K*α radiation, λ = 0.71073 Å
Hall symbol: -P 2yn	Cell parameters from 5671 reflections
*a* = 7.0655 (2) Å	θ = 2.3–29.2°
*b* = 26.7603 (7) Å	µ = 2.05 mm^−^^1^
*c* = 9.5220 (2) Å	*T* = 100 K
β = 94.554 (2)°	Prism, colorless
*V* = 1794.69 (8) Å^3^	0.30 × 0.25 × 0.20 mm
*Z* = 4	

### Data collection

**Table:**

Agilent SuperNova Dual diffractometer with an Atlas detector	3964 independent reflections
Radiation source: SuperNova (Mo) X-ray Source	3582 reflections with *I* > 2σ(*I*)
Mirror	*R*_int_ = 0.024
Detector resolution: 10.4041 pixels mm^-1^	θ_max_ = 27.5°, θ_min_ = 2.3°
ω scans	*h* = −7→9
Absorption correction: multi-scan (*CrysAlis PRO*; Agilent Technologies, 2010)	*k* = −19→33
*T*_min_ = 0.579, *T*_max_ = 0.685	*l* = −12→11
8726 measured reflections	

### Refinement

**Table:**

Refinement on *F*^2^	Primary atom site location: structure-invariant direct methods
Least-squares matrix: full	Secondary atom site location: difference Fourier map
*R*[*F*^2^ > 2σ(*F*^2^)] = 0.025	Hydrogen site location: inferred from neighbouring sites
*wR*(*F*^2^) = 0.059	H-atom parameters constrained
*S* = 1.02	*w* = 1/[σ^2^(*F*_o_^2^) + (0.0273*P*)^2^ + 1.1852*P*] where *P* = (*F*_o_^2^ + 2*F*_c_^2^)/3
3964 reflections	(Δ/σ)_max_ = 0.001
228 parameters	Δρ_max_ = 0.58 e Å^−^^3^
0 restraints	Δρ_min_ = −0.71 e Å^−^^3^

### Fractional atomic coordinates and isotropic or equivalent isotropic displacement parameters (Å^2^)

**Table:**

	*x*	*y*	*z*	*U*_iso_*/*U*_eq_	
Sn1	0.60924 (2)	0.655455 (6)	0.329618 (16)	0.01294 (6)	
Cl1	0.37306 (8)	0.67760 (2)	0.14686 (6)	0.01817 (13)	
Cl2	0.86908 (9)	0.70312 (3)	0.26342 (7)	0.02392 (15)	
Cl3	0.50421 (8)	0.72003 (2)	0.48125 (6)	0.01835 (13)	
Cl4	0.70745 (9)	0.58382 (2)	0.20077 (6)	0.02003 (14)	
O1	0.7650 (2)	0.62593 (7)	0.50542 (17)	0.0159 (4)	
O2	0.7745 (3)	0.56931 (7)	0.67716 (18)	0.0218 (4)	
O3	1.1503 (3)	0.55738 (7)	1.01670 (19)	0.0237 (4)	
O4	1.4080 (3)	0.57009 (8)	0.89617 (19)	0.0265 (4)	
N1	0.4045 (3)	0.60323 (8)	0.4241 (2)	0.0137 (4)	
N2	0.2115 (3)	0.52822 (8)	0.5541 (2)	0.0189 (5)	
N3	0.8601 (3)	0.67062 (8)	0.8236 (2)	0.0157 (4)	
N4	1.2150 (3)	0.64576 (8)	0.7473 (2)	0.0194 (5)	
C1	0.6920 (3)	0.59043 (9)	0.5772 (2)	0.0151 (5)	
C2	0.4893 (3)	0.57708 (9)	0.5319 (2)	0.0135 (5)	
C3	0.3911 (4)	0.53973 (9)	0.5957 (2)	0.0167 (5)	
H3	0.4537	0.5217	0.6717	0.020*	
C4	0.1292 (3)	0.55520 (10)	0.4485 (3)	0.0187 (5)	
H4	0.0009	0.5483	0.4172	0.022*	
C5	0.2236 (3)	0.59312 (10)	0.3823 (3)	0.0170 (5)	
H5	0.1597	0.6117	0.3079	0.020*	
C6	1.2439 (4)	0.51458 (11)	1.0871 (3)	0.0283 (6)	
H6A	1.1620	0.5007	1.1558	0.042*	
H6B	1.3646	0.5253	1.1357	0.042*	
H6C	1.2680	0.4890	1.0172	0.042*	
C7	1.2504 (4)	0.58058 (10)	0.9240 (3)	0.0190 (5)	
C8	1.1371 (3)	0.62241 (10)	0.8531 (2)	0.0161 (5)	
C9	0.9594 (3)	0.63438 (10)	0.8935 (3)	0.0171 (5)	
H9	0.9082	0.6173	0.9693	0.021*	
C10	0.9332 (4)	0.69445 (10)	0.7175 (3)	0.0178 (5)	
H10	0.8632	0.7200	0.6675	0.021*	
C11	1.1126 (4)	0.68141 (10)	0.6813 (3)	0.0201 (5)	
H11	1.1643	0.6987	0.6060	0.024*	
C12	0.6682 (3)	0.68313 (11)	0.8634 (3)	0.0225 (6)	
H12A	0.6061	0.7056	0.7928	0.034*	
H12B	0.6777	0.6996	0.9556	0.034*	
H12C	0.5933	0.6525	0.8684	0.034*	

### Atomic displacement parameters (Å^2^)

**Table:**

	*U*^11^	*U*^22^	*U*^33^	*U*^12^	*U*^13^	*U*^23^
Sn1	0.01198 (9)	0.01295 (10)	0.01393 (9)	−0.00115 (6)	0.00119 (6)	0.00060 (6)
Cl1	0.0190 (3)	0.0184 (3)	0.0166 (3)	0.0002 (2)	−0.0016 (2)	0.0015 (2)
Cl2	0.0176 (3)	0.0224 (4)	0.0324 (3)	−0.0056 (3)	0.0063 (3)	0.0047 (3)
Cl3	0.0208 (3)	0.0170 (3)	0.0172 (3)	0.0022 (2)	0.0015 (2)	−0.0020 (2)
Cl4	0.0217 (3)	0.0188 (3)	0.0199 (3)	0.0023 (2)	0.0029 (2)	−0.0030 (2)
O1	0.0132 (8)	0.0157 (9)	0.0180 (8)	−0.0006 (7)	−0.0036 (7)	0.0014 (7)
O2	0.0225 (9)	0.0220 (11)	0.0200 (9)	−0.0004 (8)	−0.0048 (7)	0.0033 (8)
O3	0.0239 (10)	0.0209 (11)	0.0259 (10)	0.0029 (8)	−0.0005 (8)	0.0048 (8)
O4	0.0220 (10)	0.0319 (12)	0.0253 (10)	0.0089 (8)	0.0009 (8)	−0.0018 (8)
N1	0.0120 (9)	0.0124 (11)	0.0169 (10)	−0.0008 (8)	0.0017 (8)	−0.0009 (8)
N2	0.0182 (10)	0.0157 (12)	0.0230 (11)	−0.0018 (9)	0.0030 (9)	0.0006 (9)
N3	0.0156 (10)	0.0153 (11)	0.0159 (10)	−0.0013 (8)	−0.0009 (8)	−0.0031 (8)
N4	0.0159 (10)	0.0218 (13)	0.0204 (11)	−0.0029 (9)	0.0006 (9)	−0.0014 (9)
C1	0.0188 (12)	0.0119 (13)	0.0143 (11)	0.0015 (10)	−0.0010 (10)	−0.0027 (9)
C2	0.0158 (11)	0.0114 (12)	0.0135 (11)	0.0025 (9)	0.0022 (9)	−0.0007 (9)
C3	0.0223 (12)	0.0133 (13)	0.0147 (11)	0.0003 (10)	0.0025 (10)	−0.0008 (10)
C4	0.0133 (11)	0.0186 (14)	0.0243 (13)	−0.0015 (10)	0.0021 (10)	−0.0018 (11)
C5	0.0154 (11)	0.0171 (14)	0.0183 (12)	0.0007 (10)	0.0002 (10)	0.0006 (10)
C6	0.0344 (16)	0.0207 (16)	0.0282 (14)	0.0040 (12)	−0.0068 (12)	0.0027 (12)
C7	0.0194 (13)	0.0199 (14)	0.0172 (12)	−0.0016 (11)	−0.0025 (10)	−0.0052 (10)
C8	0.0164 (12)	0.0149 (13)	0.0165 (12)	−0.0015 (10)	−0.0012 (10)	−0.0036 (9)
C9	0.0182 (12)	0.0158 (13)	0.0168 (12)	−0.0006 (10)	−0.0010 (10)	−0.0013 (10)
C10	0.0198 (12)	0.0145 (13)	0.0187 (12)	−0.0020 (10)	−0.0019 (10)	−0.0008 (10)
C11	0.0214 (13)	0.0198 (15)	0.0191 (12)	−0.0054 (11)	0.0018 (10)	−0.0004 (11)
C12	0.0147 (12)	0.0246 (16)	0.0284 (14)	0.0036 (11)	0.0037 (11)	−0.0007 (12)

### Geometric parameters (Å, °)

**Table:**

Sn1—O1	2.0843 (17)	N4—C8	1.340 (3)
Sn1—N1	2.2499 (19)	C1—C2	1.506 (3)
Sn1—Cl2	2.3619 (6)	C2—C3	1.384 (3)
Sn1—Cl1	2.3881 (6)	C3—H3	0.9500
Sn1—Cl3	2.4065 (6)	C4—C5	1.392 (4)
Sn1—Cl4	2.4076 (6)	C4—H4	0.9500
O1—C1	1.301 (3)	C5—H5	0.9500
O2—C1	1.215 (3)	C6—H6A	0.9800
O3—C7	1.328 (3)	C6—H6B	0.9800
O3—C6	1.459 (3)	C6—H6C	0.9800
O4—C7	1.199 (3)	C7—C8	1.504 (4)
N1—C5	1.336 (3)	C8—C9	1.380 (3)
N1—C2	1.343 (3)	C9—H9	0.9500
N2—C4	1.333 (3)	C10—C11	1.384 (4)
N2—C3	1.335 (3)	C10—H10	0.9500
N3—C10	1.333 (3)	C11—H11	0.9500
N3—C9	1.342 (3)	C12—H12A	0.9800
N3—C12	1.475 (3)	C12—H12B	0.9800
N4—C11	1.326 (3)	C12—H12C	0.9800
			
O1—Sn1—N1	76.03 (7)	N2—C4—C5	122.7 (2)
O1—Sn1—Cl2	92.70 (5)	N2—C4—H4	118.6
N1—Sn1—Cl2	168.60 (5)	C5—C4—H4	118.6
O1—Sn1—Cl1	166.67 (5)	N1—C5—C4	119.6 (2)
N1—Sn1—Cl1	90.65 (5)	N1—C5—H5	120.2
Cl2—Sn1—Cl1	100.63 (2)	C4—C5—H5	120.2
O1—Sn1—Cl3	87.62 (5)	O3—C6—H6A	109.5
N1—Sn1—Cl3	88.13 (5)	O3—C6—H6B	109.5
Cl2—Sn1—Cl3	93.19 (2)	H6A—C6—H6B	109.5
Cl1—Sn1—Cl3	91.61 (2)	O3—C6—H6C	109.5
O1—Sn1—Cl4	87.27 (5)	H6A—C6—H6C	109.5
N1—Sn1—Cl4	85.97 (5)	H6B—C6—H6C	109.5
Cl2—Sn1—Cl4	91.86 (2)	O4—C7—O3	126.2 (3)
Cl1—Sn1—Cl4	92.25 (2)	O4—C7—C8	123.1 (2)
Cl3—Sn1—Cl4	172.98 (2)	O3—C7—C8	110.7 (2)
C1—O1—Sn1	119.54 (15)	N4—C8—C9	122.6 (2)
C7—O3—C6	115.3 (2)	N4—C8—C7	116.6 (2)
C5—N1—C2	118.7 (2)	C9—C8—C7	120.8 (2)
C5—N1—Sn1	129.58 (17)	N3—C9—C8	118.7 (2)
C2—N1—Sn1	111.54 (15)	N3—C9—H9	120.6
C4—N2—C3	116.5 (2)	C8—C9—H9	120.6
C10—N3—C9	120.2 (2)	N3—C10—C11	119.1 (2)
C10—N3—C12	120.4 (2)	N3—C10—H10	120.5
C9—N3—C12	119.4 (2)	C11—C10—H10	120.5
C11—N4—C8	116.8 (2)	N4—C11—C10	122.6 (2)
O2—C1—O1	124.5 (2)	N4—C11—H11	118.7
O2—C1—C2	120.0 (2)	C10—C11—H11	118.7
O1—C1—C2	115.5 (2)	N3—C12—H12A	109.5
N1—C2—C3	120.2 (2)	N3—C12—H12B	109.5
N1—C2—C1	116.9 (2)	H12A—C12—H12B	109.5
C3—C2—C1	122.9 (2)	N3—C12—H12C	109.5
N2—C3—C2	122.3 (2)	H12A—C12—H12C	109.5
N2—C3—H3	118.9	H12B—C12—H12C	109.5
C2—C3—H3	118.9		
			
N1—Sn1—O1—C1	6.32 (17)	O1—C1—C2—C3	179.1 (2)
Cl2—Sn1—O1—C1	−171.93 (17)	C4—N2—C3—C2	−1.1 (4)
Cl1—Sn1—O1—C1	8.0 (3)	N1—C2—C3—N2	0.0 (4)
Cl3—Sn1—O1—C1	94.98 (17)	C1—C2—C3—N2	−179.8 (2)
Cl4—Sn1—O1—C1	−80.20 (17)	C3—N2—C4—C5	0.8 (4)
O1—Sn1—N1—C5	178.9 (2)	C2—N1—C5—C4	−1.6 (4)
Cl2—Sn1—N1—C5	−172.28 (19)	Sn1—N1—C5—C4	173.07 (17)
Cl1—Sn1—N1—C5	−0.7 (2)	N2—C4—C5—N1	0.5 (4)
Cl3—Sn1—N1—C5	90.9 (2)	C6—O3—C7—O4	−1.4 (4)
Cl4—Sn1—N1—C5	−92.9 (2)	C6—O3—C7—C8	177.4 (2)
O1—Sn1—N1—C2	−6.18 (15)	C11—N4—C8—C9	−0.2 (4)
Cl2—Sn1—N1—C2	2.7 (4)	C11—N4—C8—C7	177.6 (2)
Cl1—Sn1—N1—C2	174.22 (15)	O4—C7—C8—N4	5.1 (4)
Cl3—Sn1—N1—C2	−94.20 (15)	O3—C7—C8—N4	−173.7 (2)
Cl4—Sn1—N1—C2	82.01 (15)	O4—C7—C8—C9	−177.1 (2)
Sn1—O1—C1—O2	176.53 (19)	O3—C7—C8—C9	4.1 (3)
Sn1—O1—C1—C2	−5.3 (3)	C10—N3—C9—C8	−0.1 (4)
C5—N1—C2—C3	1.4 (3)	C12—N3—C9—C8	178.7 (2)
Sn1—N1—C2—C3	−174.21 (18)	N4—C8—C9—N3	0.4 (4)
C5—N1—C2—C1	−178.8 (2)	C7—C8—C9—N3	−177.3 (2)
Sn1—N1—C2—C1	5.6 (3)	C9—N3—C10—C11	−0.3 (4)
O2—C1—C2—N1	177.5 (2)	C12—N3—C10—C11	−179.1 (2)
O1—C1—C2—N1	−0.7 (3)	C8—N4—C11—C10	−0.2 (4)
O2—C1—C2—C3	−2.7 (4)	N3—C10—C11—N4	0.5 (4)

## References

[bb1] Agilent Technologies (2010). *CrysAlis PRO* Agilent Technologies, Yarnton, England.

[bb2] Barbour, L. J. (2001). *J. Supramol. Chem.* **1**, 189–191.

[bb3] Ma, C.-L., Han, Y.-F., Zhang, R.-F. & Wang, D.-Q. (2004). *Dalton Trans.* pp. 1832–1840. 10.1039/b404477k 15381988

[bb4] Sheldrick, G. M. (2008). *Acta Cryst.* A**64**, 112–122. 10.1107/S0108767307043930 18156677

[bb5] Westrip, S. P. (2010). *J. Appl. Cryst.* **43**, 920–925.

